# Pharmacological blockade of TNFα prevents sarcopenia and prolongs survival in aging mice

**DOI:** 10.18632/aging.202200

**Published:** 2020-11-26

**Authors:** Clara Sciorati, Riccardo Gamberale, Antonella Monno, Lorena Citterio, Chiara Lanzani, Rebecca De Lorenzo, Giuseppe A. Ramirez, Antonio Esposito, Paolo Manunta, Angelo A. Manfredi, Patrizia Rovere-Querini

**Affiliations:** 1 Division of Immunology, Transplantation and Infectious Diseases, IRCCS Ospedale San Raffaele Scientific Institute, Milan, Italy; 2 Unit of Nephrology, IRCCS Ospedale San Raffaele, Milan, Italy; 3 Vita-Salute San Raffaele University, Milan, Italy; 4 Experimental Imaging Centre, San Raffaele Scientific Institute, Milan, Italy

**Keywords:** sarcopenia, aging, TNF alpha, inflammation, pharmacological intervention

## Abstract

Sarcopenia is a hallmark of aging. Inflammation due to increased generation of cytokines such as TNFα, IL-1β and IL-6 has been implicated in the pathogenesis of sarcopenia. In skeletal muscle of C57BL/6 mice from 12 until 28 months of age, we observed a progressive reduction of myofiber cross sectional area, loss of type II fibers and infiltration by inflammatory cells. Muscle strength decreased in parallel. Pharmacological TNFα blockade by weekly subcutaneous injection of Etanercept from 16 to 28 months of age prevented atrophy and loss of type II fibers, with significant improvements in muscle function and mice lifespan. The effects on leukocyte recruitment were limited. These results provide a proof of principle that endogenous TNFα is sufficient to cause sarcopenia and to reduce animal survival, and open a novel perspective on novel potential pharmacological treatment strategies based on TNFα blockade to prevent the noxious events associated with aging.

## INTRODUCTION

Aging-related sarcopenia is a process characterized by loss of skeletal muscle mass and function, with an increased risk of adverse outcomes [1, 2]. Sarcopenia is characterized by a progressive reduction of the number and diameter of myofibers, with loss of motor units and a change in the ratio of type I to type II fibers [3, 4]. Mechanisms leading to sarcopenia include satellite cell and mitochondrial dysfunction, unbalance between muscle protein synthesis and breakdown, neuromuscular junction degeneration and vessel defects [1]. However, little is known about the causes of these age-associated changes.

Aging is accompanied by chronic low-grade inflammation (“inflammaging”) [5], which could have a causal role in sarcopenia [6]. Tumor necrosis factor-α (TNFα), interleukin-6 (IL-6), interleukin-10 and interleukin-15 might contribute to the loss of muscle mass [7, 8]. TNFα is a particularly interesting candidate, being a non-redundant target in inflammatory human diseases associated to complex multi-cytokine inflammatory responses. In addition, TNFα is known to promote muscle wasting and cachexia [9] by promoting protein degradation while decreasing protein synthesis [10, 11] and by inhibiting muscle regeneration by blocking proliferation and differentiation of muscle stem cells [12]. In aged muscle, this inhibition seems to be preferentially mediated by TNFα released by bone marrow-derived leukocytes and TNFα genetic knockdown protects against aging-induced fiber loss and reduction of stem cell regenerative capacity [13]. TNFα also promotes apoptosis of both type I and type II muscle fibers [14].

Etanercept is a dimeric fusion protein consisting of the extracellular portion of the human TNFα receptor 75-kilodaltons (p75) bound to the human IgG1 Fc moiety. Etanercept acts as soluble receptor interfering with TNFα binding to tissue receptors and has been employed for the treatment of autoimmune diseases for over two decades, with excellent efficacy and safety profiles [15, 16]. We evaluated the effects of TNFα blockade on spontaneous aging in wild type mice aged 16 to 28 months (corresponding to 50-90 years of human age) [17]. This treatment quenched age-associated spontaneous muscle loss, reduced fiber type shift, improved muscle function and increased animal life span.

## RESULTS

### TNFα blockade prevents skeletal age-related muscle atrophy and loss of function and prolongs lifespan

**Figure 1:**
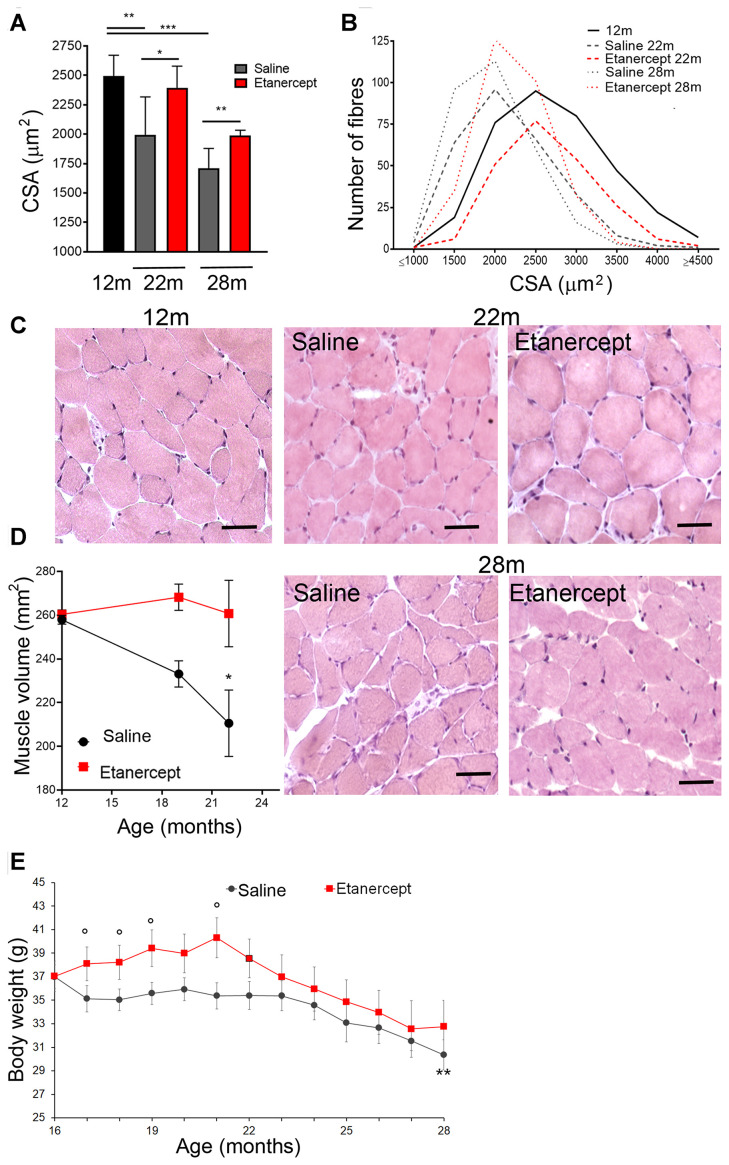
**Blockade of TNFα by Etanercept inhibited sarcopenia during spontaneous aging.** Measurement of cross-sectional area (CSA) (**A**) and fiber size distribution (**B**) in GS of 12 months old mice and in control (saline) or Etanercept-treated mice at 22 and 28 months of age. *=p<0.05, **= p<0.01 and *** =p<0.001. (**C**) Representative histochemical images of GS muscle sections analyzed in 12 months old mice and in control or Etanercept-treated mice at 22 and 28 months of age. Scale bar=50 μm (magnification of the16x) (**D**). MRI measurement of muscle volume for control or Etanercept-treated mice at 12, 19 and 22 months of age. *=p<0.05 in comparison to 12 months of age (**E**) Body weight of control and Etanercept-treated mice monitored between 16 and 28 months of age. ** =p<0.01 vs 16 months of age, °= p<0.05 vs weight of control mice measured at the same time point. Data are show as mean ± standard error.

We measured the cross-sectional area (CSA) of gastrocnemius (GS) muscle fibers at 12, 22 and 28 months of age. Mean CSA significantly decreased at 22 and 28 months of age in mice (Figure 1A), reflecting the physiological age-associated atrophy. The number of smaller fibers increased in the muscle of older mice (Figure 1B). TNFα blockade (starting from 16 months of age) limited atrophy, as demonstrated by comparing the CSA of mice treated with Etanercept or saline (control) at 22 and 28 months of age (*i.e.* after 6 and 12 months of treatment, respectively; Figure 1A). Conversely, the number of smaller fibers was lower in mice treated with Etanercept as compared with controls (Figure 1B). Representative H&E images are shown in Figure 1C. Results obtained by morphometry were confirmed by measurement of muscle volume by magnetic resonance imaging (MRI) (Figure 1D). Body weight of control mice also progressively decreased with age. Mice treated with Etanercept were significantly heavier in the first phase of treatment (five months). The effect was not detectable at later timepoints (Figure 1E).

**Figure 2:**
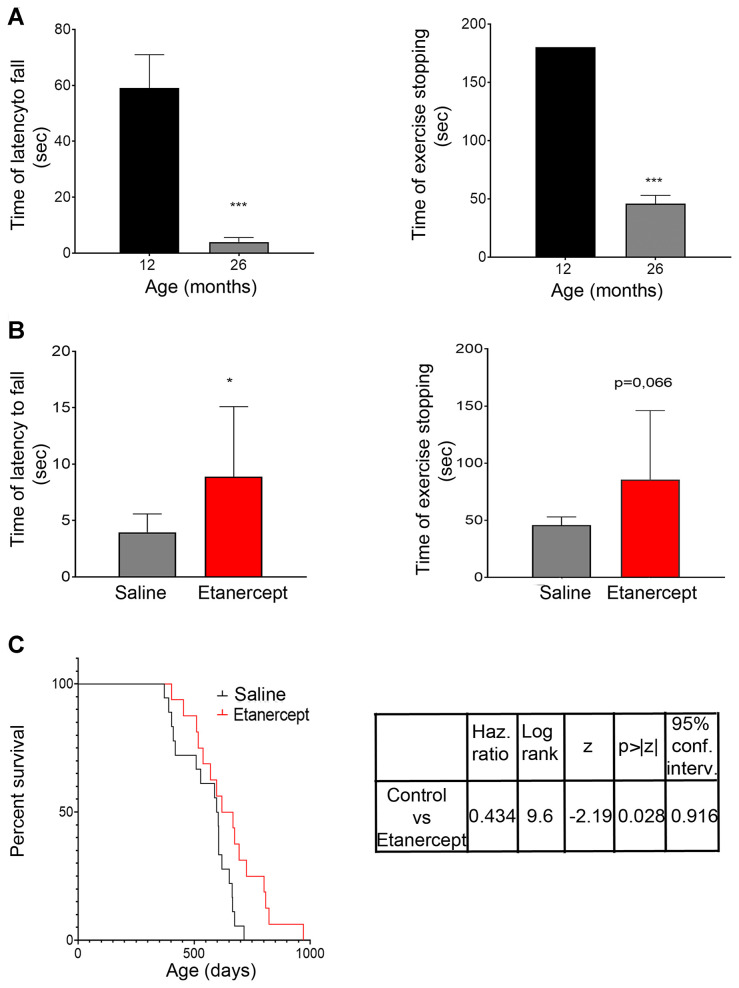
**TNFα blockade inhibited muscle functional impairment during spontaneous aging and increased lifespan.** (**A**) Mice performance during hanging wire test evaluated at 12 and 26 months of age. Both average times spent on wire between each fall (referred to as latency to fall) and the time required to complete the exercise (referred to as time of exercise stopping) were measured. ***= p<0.001 vs 12 months; n≥9 mice/age (**B**) Measurements obtained for control (saline) or Etanercept-treated mice evaluated at 26 months of age. *= p<0.05 vs control mice, n≥9 mice/group. (**C**) Kaplan-Meyer survival curve comparing control and Etanercept-treated mice.

To assess the functional consequences of skeletal muscle loss, muscle strength was assessed using the hanging wire test, a validated method that takes into account the average latency to fall off a wire (“*latency to fall*”) and the time needed to complete the exercise (“*time of exercise stopping”*). Untreated aging mice showed a spontaneous decline in muscle strength (Figure 2A), which was prevented by TNF-blockade (Figure 2B). Furthermore, control animals spontaneously died within 700 days (≈ 23 months), whereas mice treated with anti-TNFα showed a longer lifespan (Figure 2C).

### Blockade of TNFα prevents micro-architectural involution of skeletal muscle

**Figure 3:**
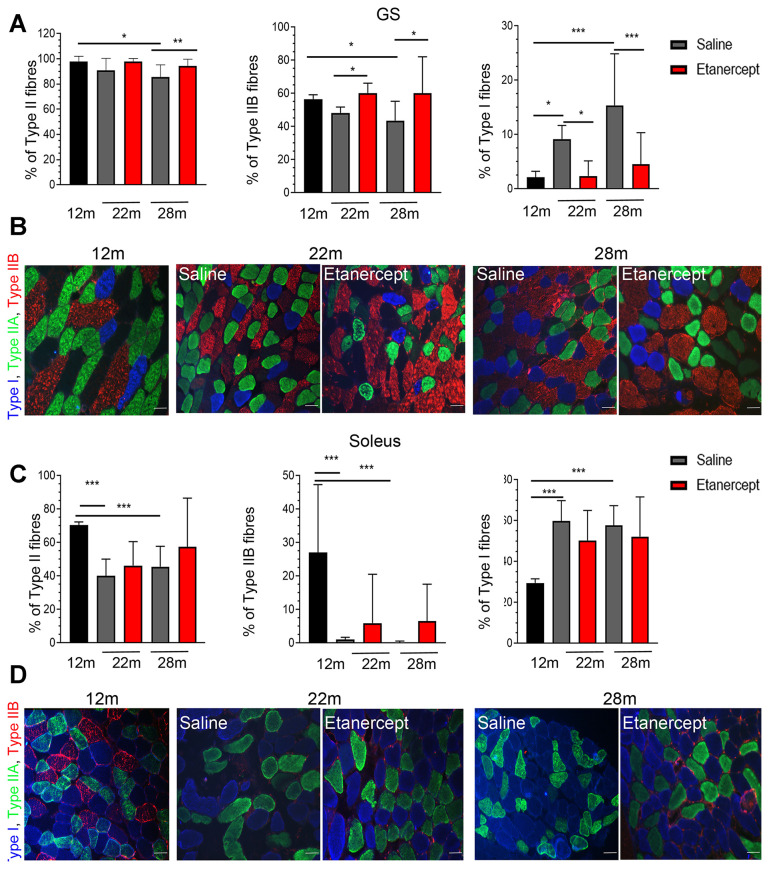
**TNFα blockade inhibited the loss of type II fast twitch fibers.** (**A**) Percentage of Type II (left), Type IIB (middle) and Type I (right) fibers in GS muscles of 12 months old mice in controls (saline) or Etanercept-treated mice at 22 and 28 months of age, *=p<0.05, **= p<0.01 and ***=p<0.001. (**B**) Representative images of GS muscle sections. Type I fibers are blue, type IIA are green, type IIB are red. Scale bar=50 μm. (**C**) Percentage of Type II (left), Type IIB (middle) and Type I (right) fibers in soleus muscles of 12 months old mice or in control or Etanercept-treated mice at 22 and 28 months of age. *=p<0.05, **= p<0.01. (**D**) Representative images of soleus muscle sections. Scale bar=50 μm.

**Figure 4:**
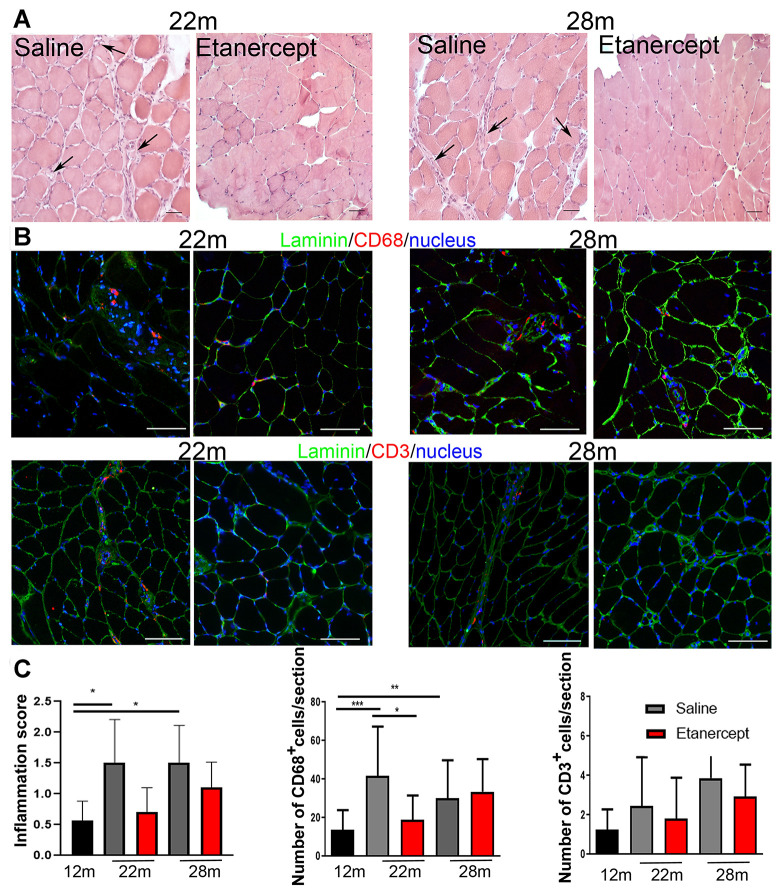
**TNFα blockade reduced inflammation.** (**A**) Representative images of muscle sections analyzed at 22 or 28 months of age in control (saline) and Etanercept-treated mice. Scale bar=50 μm. Arrows indicate perivascular inflamed areas. (**B**) Representative images of muscle CD68- and CD3-positive cells (both in red) in control and Etanercept-treated muscle sections at 22 and 28 months of age. Laminin staining (green) identifies fibers while cell nuclei are blue. Scale bar=50 μm (magnification of 16x acquisition). (**C**) Inflammation score and CD68^+^ and CD3^+^ cell number evaluated in 12 months old mice and in control and Etanercept-treated mice at 22 and 28 months of age.

Type I (slow twitch) and type II (fast twitch) fibers in GS and *soleus* muscles were identified at 12, 22 and 28 months of age by immunofluorescence. The fraction of type II fibers decreased with age, with fully glycolytic type IIB fibers undetectable in *soleus* muscle sections as early as 22 weeks of age. Conversely, type I red, oxidative fibers increased (Figure 3A, 3C). Blockade of TNFα prevented tissue reorganization and reduction in type II fiber (including IIB) at both 22 and 28 months of age in GS (Figure 3A). Representative images of the GS and *soleus* muscles are shown in panels B and D of Figure 3. To assess the overall muscle changes, mice were studied by MRI at 12, 19, 22 months of age measuring fractional anisotropy (FA) and T2 relaxation time (T2-rt) [18]. FA, which increases when muscle architectural reorganization interferes with the anisotropic property of the muscle causing loss of direction of water molecules, increased significantly from month 12 (0.249 ±0.0042) to month 19 (0.31±0.007; p<0.01) and month 22 (0.33±0.01; p<0.001) in control mice. TNFα blockade lowered FA at 22 months of age (0.314±0.005 vs 0.33±0.01 p<0.05). No significant changes were detected in T2-rt, which reflects muscle edema and is observed in acute injury and infection (not shown). Muscle histology revealed low-grade inflammation, primarily in perivascular areas (Figure 4A, arrows). Inflammatory cells mainly consisted of macrophages (CD68+ cells), while lymphocyte-infiltrated areas (CD3+ cells) were rare (Figure 4B). Etanercept had limited effects on the extent of inflammatory cell infiltration (Figure 4C). Specifically, TNFα blockade reduced the number of infiltrating macrophages at 22 months of age (Figure 4, C) but the effect was not detectable at 28 months. Moreover, the number of lymphocytes infiltrating the tissue did not significantly change. A modest but significant increase in serum levels of IL-6 and of granulocyte colony-stimulating factor (G-CSF) was observed at month 22 compared to month 12 of age (IL-6: 92.79 ± 82.71 pg/ml vs 2.01 ± 1.24 pg/ml; G-CSF: 557.84 ± 388.68 vs 107.50 ± 49.69 pg/ml). TNFα blockade dampened cytokine production (IL-6: 35.38± 48.15 pg/ml vs 92.79 ± 82.71; G-CSF: 125.41± 88.718 vs 557.84 ± 388.68 pg/ml).

## DISCUSSION

Age-related sarcopenia is associated with a reduction in the number and size of muscle fibers and causes a progressive decline in muscle function [1]. Chronic low-grade systemic inflammation might contribute to sarcopenia [5, 6].

The muscle is increasingly recognized as an organ with immune regulatory properties exerted by the secretion of myokines (such as IL-6, IL-7, IL-15) or through cell-cell interactions [19, 20]. The regenerative potential of skeletal muscle depends on its interactions with immune cells infiltrating damaged tissue to remove debris and promote satellite cell function [21]. The equilibrium of these interactions could be lost with aging.

We found that spontaneous aging in mice reflects many features of human aging at the skeletal muscle level. Specifically, we observed that over an observation window of 16 months (between 12 and 28 months of age, corresponding to 40-90 years of age in humans) [17], aging mice lost weight and muscle mass. This was accompanied by progressive reduction of fiber CSA and of mouse endurance during exercise. Fiber size decline from 12 to 22 months was severe and further worsened at 28 months. Accordingly, MRI-measured muscle volume and body weight decreased starting from month 22 of age. The cells of innate and acquired immunity were consistently detectable in skeletal muscle at 22 months of age and remained stable thereafter. The concentration of circulating cytokines followed comparable kinetics. This suggests that inflammation could start early in aged mice, reaching a “steady state” at a low level for an extended period. This could be associated with the functional defects observed in senescent macrophages [22].

In humans, vessel alterations have been described in aging [23]. Such changes in vascular homeostasis may affect muscle metabolism, fitness and function through decreased oxygen supply and impaired delivery of nutrients and hormones [24]. The most striking feature we observed is a severe reduction in the percentage of type II fast twitch fibers and the increase in type I fibers, which is consistent with previous observations in aging humans [25]. Fully glycolytic type II B fibers were particularly affected and had already completely disappeared in *soleus* at 22 months of age. Skeletal muscle fibers are differentially sensitive to environmental signals. Red, oxidative type I fibers have a higher rate of protein synthesis and degradation and are more resistant to fasting. Conversely, they are more sensitive to inactivity and denervation. Fast fibers, on the other hand, are strongly affected by protein breakdown [26]. This suggests that regulation of the protein synthesis/degradation rate could be a key mechanism of age-induced sarcopenia.

Interventions aimed to maintain muscle function could be important for improving age-induced sarcopenia. Cyclooxygenase inhibitors improve mass and strength in older muscle when used in combination with resistance training programs [27]. IL-6 antagonists have been proposed for the treatment of cachexia in patients with cancer [28] or rheumatoid arthritis [29]. Anti-TNFα antibodies might also control muscle loss in chronic diseases such as Crohn's disease and rheumatic diseases [30, 31]. However, these observations are difficult to interpret, as other effects associated to anti-TNFα treatment might contribute in these patients, including corticosteroid tapering allowed by better control of the inflammatory state [32].

A causal link between chronic inflammation in skeletal muscle and sarcopenia has not yet been established. In skeletal muscle, exogenous TNFα, also known as cachectin, promotes tissue loss and impairs muscle function [9]. TNFα also down-regulates the expression of anabolic hormones and growth factors [33], reduces the expression of MyoD and myogenin in regenerating muscles and increases MyoD degradation through the ubiquitin proteasome pathway in myoblasts [12, 34]. Genetic ablation of TNFα, which is mainly expressed by myeloid cells in skeletal muscle, reduces sarcopenia and improves satellite cell activation in aged mice [13].

We investigated whether pharmacological blockade of TNFα by a well-established agent (Etanercept) might affect sarcopenia in aging mice. The treatment was carried out for 12 months, from month 16 to month 28 of age, corresponding to 50-80 years of human age [17]. The drug is a dimeric soluble form of the p75 receptor that inhibits TNFα by blocking its interaction with cell-surface TNFα receptors. Specifically, Etanercept is the extracellular ligand-binding portion of the human p75 receptor linked to the Fc portion of human IgG1 [35]. The drug was administered subcutaneously using a dose that effectively blocks the cytokine-elicited NF-κB activation *in vitro* (*not shown*) and inhibits muscle damage in mouse models of limb-girdle and of Duchenne dystrophies, and of inflammatory idiopathic myopathies [36–39]. The administration schedule (once per week) was chosen to reduce the development of neutralizing anti-drug antibodies (due to its human origin) [40], which are known to diminish the clinical response and might be favored by the length of the treatment (12 months).

Treatment with Etanercept reduced sarcopenia by limiting the decrease in CSA, preserved body weight and muscular mass and prevented loss of strength and endurance in aged mice. Muscle volume did not statistically change between 12 and 22 months of age in treated mice, while it decreased in control animals. Protection against atrophy was higher at 22 in comparison to 28 months of age (16.9% ± 7.4 and 11.2% ± 1.8 respectively). Similarly, body weight was maintained in treated mice, whereas a decrease was observed in control animals between 16 and 28 months of age. The relative reduction of Etanercept efficacy after several months of treatment could be explained by the human origin of the agent [35] and the partial development of blocking antibodies in mice [40, 41]. The effect of Etanercept on atrophy could be a result of improved satellite cell activation and increased regeneration. In support, the genetic ablation of TNFα resulted in reduced Pax7 expression and in an increased number of centronucleated regenerating fibers, while isolated myoblasts are more fusogenic [13].

We found that treatment with Etanercept preserved muscle strength, as measured by hanging wire test at 26 months of age. However, to reduce the number of mice needed to perform the experiments, animals were not evaluated for strength at the same time points as for biochemical and histological analyses, and this represents a limitation of our study. The results demonstrated that, despite possible drug inactivation mechanisms, TNFα inhibition during the early phases of atrophy development is effective in preserving muscle performance in later stages. The treatment also resulted in an increased percentage of type II fibers at 22 and 28 months of age, while it prevented type I fiber increase. This effect was more prominent in GS, in agreement with previous observations suggesting that TNFα causes greater atrophy in muscles with prevalence of type IIA and type IIB fibers [42]. Interestingly, TNFα blockade had a long-lasting preventive effect towards atrophy, type II fiber loss and muscle weakness while its anti-inflammatory effects were less evident at 28 months of age, suggesting a non-redundant role of TNFα in priming muscle architectural involution.

Treated mice also lived longer. This finding nicely fits with the link described between sarcopenia and reduced life-expectancy in humans [43] and further supports the usefulness of the spontaneous model of aging in normal mice we used in these experiments. With all the limitations associated with preclinical studies, our findings suggest that this approach could have clinical applications, as Etanercept is safe and well tolerated in the elderly [44]. Further studies are necessary to evaluate whether Etanercept should be considered for the prevention or treatment of aging-induced sarcopenia.

## MATERIALS AND METHODS

### Animals and treatment

Wild-type female C57/BL6 mice (Charles River Laboratories, Lecco, Italy) were housed in the pathogen-free facility at our Institution at 12 months of age and left aging spontaneously in accordance with the European Community guidelines and with the approval of the Institutional Ethical Committee (IACUC authorization number 842). Mice (23 animals/group) received weekly subcutaneous injection of either Etanercept (Enbrel, 1 mg/kg in physiologic solution Pfizer, NY, USA) or vehicle (saline) from 16 months to 28 months of age. Spontaneous development of kyphosis, occurrence of skin lesions and neoplasia, weight loss, lethargy, breathing difficulties, impaired movement and alopecia were recorded [17]. Animals were evaluated initially weekly and then daily and euthanized at study timepoints.

### Inflammation grading and fiber morphometry

Five mice/group were euthanized at 12, 22 and 28 months of age (*i.e.* either before and 6 or 12 months after the beginning of treatment). Right and left *gastrocnemius* (GS) and *soleus* muscles were isolated, immediately frozen in liquid nitrogen cooled isopentane and stored at -80°. Half of the muscles were cut, taking care to place on the histological slides obtained for a sample, only the slices that had the same position in the muscle. At least ten 8-10 μm-thick slices for each slide were assessed. Sections stained with hematoxylin and eosin were used for inflammation score and fiber CSA after image acquisition by Axioskop microscope (Zeiss NY, USA). At least five representative photos were obtained for each muscle using adequate magnification. CSA was determined by ImageJ software using a 100 μm scale as reference and measuring at least 250 fibers for each mouse. We calculated both mean and fiber area distribution (number of fibers showing CSA < 1000, 1000-1500, 1500-2000, 2000-2500, 2500-3000, 3000-3500, 3500-4000, > 4000 μm^2^). The inflammatory infiltrate was scored under microscope by two independent expert researchers as follows: 0 = no inflammatory cells; 1 = sparse inflammatory cells or small aggregates; 2 = one or more large inflammatory cell aggregates per section.

### Immunofluorescence

For macrophages and T lymphocytes evaluation in the inflammatory infiltrate, we analyzed the expression of CD68 and CD3 by immunofluorescence. Slides were fixed with paraformaldehyde 4% for staining with anti-CD68 antibodies or frozen methanol: acetone, 1:1 solution for staining with anti-CD3 antibodies and incubated with 0.1M glycine followed by blocking buffer (5% bovine serum albumin, 5% FCS and 0.1% Triton X100 in phosphate buffer saline) for 1 hour. The slices were incubated with primary antibodies (1:100, rat anti-mouse CD68, BioRad, CA USA or 1:30, rabbit anti-mouse CD3, Abcam, Cambridge UK) overnight at 4° C and then with the appropriate secondary antibodies for 1h at RT (1:500 Alexafluor 546-coniugated rat anti-mouse or goat anti-rabbit antibodies Invitrogen CA USA). To identify skeletal muscle fibers, slices were incubated with an anti-mouse laminin antibody (1:300, chicken anti-laminin, LSbio, WA USA) followed by Alexafluor 488-coniugated anti-chicken secondary antibody (Invitrogen, CA USA). Nuclei were identified after counterstaining with Hoechst. CD68+ and CD3+ cells were quantified under the microscope by two independent expert researchers by counting the number of positive cells per slice (8-10 slices for muscle). For the assessment of the fiber type ratio, the sections were analyzed by antibodies that recognize the myosin isoforms. Type I myosin is presents in red, oxidative fibers. Type II myosin has two different subtypes, type II A, expressed in “intermeddle” fibers with an oxidative metabolism, and type II B which characterizes the white and fully glycolytic fibers. The sections were fixed in acetone, treated with blocking buffer as described and incubated with a primary antibodies cocktail (IgG1 anti-Myosin Heavy Chain Type IIA clone SC-71, IgM anti-Myosin Heavy Chain Type IIB clone BF-F3, IgG 2a anti-Myosin Heavy Chain Type I clone BA-D5, Developmental Studies Hybridoma Bank, University of Iowa, 1:70, IA USA) overnight at 4° C. Secondary antibodies (Alexafluor 488 goat anti-mouse IgG1, AlexaFluor 594 goat anti-mouse IgM, Alexa Fluor 350 goat anti-mouse IgG2b; Invitrogen, 1:500) were then added for 1 hour. Images were acquired by a Zeiss Axioskop microscope. At least five representative photos were obtained for each muscle using a 16x magnification and employed for f fiber type ratio assessment.

### Magnetic resonance imaging

MRI study was performed at 12, 18 and 22 months of age (*i.e.* before and after 3 and 6 months of treatment) by a 7 Tesla preclinical magnetic resonance scanner (BioSpec 70/30 USR, Paravision 6.0.1 Bruker, MA USA), equipped with 450/675 mT/m gradients (slow rate: 3400–4500 T/m/s; rise time: 140 μs). A phased-array rat-heart coil with four internal preamplifiers was used as receiver, coupled with a 72 mm linear-volume coil as transmitter. Mice were under general anesthesia obtained by 1.5–2% isoflurane (Forane, Abbott, IL USA) vaporized in 100% oxygen (flow: 1 l/min), in prone position, with the right leg fixed in the center of the coil. Breathing and body temperature were monitored and maintained around 30 breaths per minute and 37° C, respectively. After positioning in the magnet isocenter, a field map-based shimming (MAPSHIM software package, Paravision-5.0, Bruker) was performed to optimize field homogeneity. MRI parameters used to assess skeletal muscle included T2-relaxation time (T2-rt) and fractional anisotropy (FA). FA increases when damage and regeneration interferes with the anisotropic property of the muscle with loss of direction of water molecules while T2-rt reflects severe muscle edema and inflammation. T2-rt was calculated on muscle T2 maps obtained using a multislice-multiecho sequence with fat suppression (repetition time = 1938 ms; 16 echo times = 10.73/171.68 ms; field-of-view = 20 × 20 mm; matrix = 256 × 256; spatial resolution = 0.078 × 0.078 mm/pixel; NSA = 4) acquired on axial plane (10 slices; thickness = 1 mm; gap = 0 mm). Muscle FA was calculated on diffusion tensor images obtained using a SpinEcho-EPI sequence (DTI-Epi) with 30 diffusion gradient directions (repetition-time = 3750 ms; echo- time = 33 ms; b-values for direction = 0 sec/mm2-700 sec/mm2; diffusion gradient duration = 4 ms; diffusion gradient separation = 20 ms; NSA = 2, field-of-view = 30 x 30 mm; matrix = 128 x 128; spatial resolution = 0.234 x 0.234 mm/pixel; 10 slices; slice thickness = 1 mm; gap = 0 mm). Rare (rapid Acquisition with Relaxation Enhancement) T2 weighted images were segmented on Mipav software, (5.3.4 version, Biomedical Imaging Research Services Section, MD USA), by manual selection of areas using five slides for each mouse, to calculate muscle volume.

### Hanging wire test

We assessed muscle function by hanging wire tests performed at 12 and 26 months of age after preconditioning, as previously described [45]. Briefly, a smooth metallic plastic-coated wire (55 cm in length and 2-mm thick) was attached to two vertical supports to avoid unwanted vibrations or displacements. The wire was kept 35 cm above a layer of bedding material to prevent injury to the animal when falling. Mice were timed and endurance evaluated considering the average time spent on the wire between each fall (referred as *latency to fall)* and the time required to complete the exercise (referred as *time to exercise stopping*). The test was stopped after 3 minutes on wire or after 10 falls. The tests were repeated three times leaving at least two days between each analysis for ethical reasons and to limit animal pain due to muscular effort.

### Survival curves

Survival times were calculated from birth to spontaneous death or euthanasia for ethical reasons.

### Plasma sampling and cytokine detection

For blood sampling, the animals were pre-warmed using a red lamp for a few minutes, then blood was drawn from the tail using EDTA -treated tubes under local anesthesia. The samples were centrifuged at 13000 rpm, 4° C for 10 minutes. Plasma was collected and stored at -80° C. Cytokines were measured by commercially available sandwich ELISA tests following manufacturer’s instructions (Ab Frontiers for IL-6 and Invitrogen for G-CSF).

### Statistical analysis

GraphPad Prism 8 and STATA 15 softwares were used for statistical analysis. ANOVA and Dunnett’ post-test were used for multiple group comparison while T test, two tailed unequal variances or Mann-Whitney was employed for between-group comparisons. Data are shown as mean ± standard deviation (or standard error, when indicated). Cox regression analysis was performed to compare the survival trends between groups.
